# Pancreaticogastrostomy: A Salvage Procedure for Pancreatic Body and Neck Resection

**DOI:** 10.5812/ircmj.3112

**Published:** 2012-11-15

**Authors:** Ang Li, Pankaj Prasoon, Wu Hong, Hui Min Lu, Zhao Da Zhang, Zhang Zhaoda

**Affiliations:** 1 Department of Hepatobiliary Pancreatic Surgery, West China Hospital, Sichuan University, Sichuan Province, China

**Keywords:** Central Pancreatectomy, Pancreaticogastrostomy

## Abstract

**Abstract:**

The purpose of this analysis was to evaluate the technological viability, basic safety and consequence of central pancreatectomy (CP) with pancreaticogastrostomy in properly chosen sufferers with noncancerous central pancreatic pathology. This research is centered on the infirmary charts of West China hospital. We recruited 20 individuals from 2007 to 2009 diagnosed with benign cancerous growth of pancreatic body and neck. They underwent pancreatic body and neck resection adhering to pancreaticogastrostomy. We carried out central pancreatectomy following pancreaticogastrostomy in 20 patients: 8 with serous cyst adenomas, 11 with mucinous cystadenomas, and 1 with neuroendocrine tumor. The position of all tumors was restricted to body and neck of the pancreas, measuring a mean ± standard deviation of 2.6±1.3cm. The mean post-operative hospital stay was 7 days (ranging from 6 to 16 days).There was no intraoperative additional complications. From a technical perspective, CP is a safe and sound, pancreas-preserving pancreatectomy for non-enucleable non-cancerous pancreatic pathology restricted to the pancreatic body.

## 1. Introduction

Benign lesions of the neck and proximal body of the pancreas offer a problematic operative obstacle. If the lesions are not responsive to straightforward enucleation, specialists might be confronted with the alternative of executing pancreaticoduodenectomy or distal pancreatectomy to incorporate the lesion, ensuing in resection of a sizeable quantity of regular pancreatic parenchyma. Central pancreatic resection has been documented.

With Roux -en-Y pancreaticojejunostomy reconstruction; nevertheless, this interferes with small bowel continuity and necessitates a supplemental anastomosis. Pancreaticogastrostomy (PG) has been explored in the last several years as a substitute renovation approach when compared with pancreaticojejunostomy (PJ), being encouraged by quite a few experts (1, 2). Delcore et al. revealed a procedure of PG in which 2 to 3 cm of the pancreatic remnant was telescoped into the gastric lumen. They revealed 0% incidence of pancreatic fistula following this approach (3). Numerous prospective benefits of PG have been formerly endorsed. They incorporate the deterrence of pancreatic enzyme activation by gastric acidity and also technological facets associated to the vicinity of the pancreas to the gastric wall and diminished anastomosis in a single jejunal loop. The outstanding blood supply, the breadth of the gastric wall, and draining of the abdomen by means of nasogastric tubing suction symbolize supplemental positive aspects in favor of PG. This research is to appraisethe operative outcomes of pancreaticogastrostomy.

## 2. Case Report

Twenty sequential affected individuals underwent CP and DP from February2007 to June 2009. There were 8 women and 12 men, with the mean age of 65 ± 20 years. During their admission, almost all patients’ representing symptoms were abdominal pain, back or flank pain, along with gastroesophageal reflux disease with an unforseen finding of a pancreatic neoplasm or cyst in the course of examination to rule out their chief grievance ( Table 1 ).

**Table 1:** Demographic data of all included patients

Patients	Age	Gender	History	Main complaint	Pathology
**1**	55	M	Hypertension	Abdominal pain	Cystic neoplasm
**2**	46	F	Endometriosis	Abdominal pain	Cystic neoplasm
**3**	47	M	Hypertension/ Pneumonia	Pancreatitis	Cystic neoplasm
**4**	73	M	TCC/Afib/hypertension/CHF	Incidental	Cystic neoplasm
**5**	48	F	Hypertension/pneumonia	Pancreatitis	Cystic neoplasm
**6**	73	M	TCC/Afib a /hypertension/CHF	Incidental	Cystic neoplasm
**7**	51	F	Asthma/radiculopathy	Incidental	Cystic neoplasm
**8**	50	F	Gastroesophageal reflux disease	Incidental	Cystic neoplasm
**9**	45	M	Hypertension	Flank pain	Cystic neoplasm
**10**	63	F	Osteoarthritis/cholelithiasis	Back pain	Cystic neoplasm
**11**	38	M	None	Incidental	Cystic neoplasm
**12**	67	F	Asthma/COPD	Incidental	Cystic neoplasm
**13**	68	M	Gastro esophageal reflux disease	Incidental	Cystic neoplasm
**14**	71	M	Abdominal pain	Incidental	Cystic neoplasm
**15**	68	M	Hypertension	Incidental	Cystic neoplasm
**16**	59	M	Abdominal pain, Anxiety	Incidental	Cystic neoplasm
**17**	46	F	Abdominal pain, vomiting	Incidental	Cystic neoplasm
**18**	54	F	Osteoarthritis/cholelithiasis	Back pain	Cystic neoplasm
**19**	65	M	Hypertension, CCF ^a^	Incidental	Cystic neoplasm
**20**	59	M	Abdominal pain	Incidental	Cystic neoplasm

^a^Abbreviations: A fib: Atrial fibrillation; CAD: coronary artery disease; CCF: congestive cardiac failure

In order to guideline their chief complaints, two of them had been suspected of having mlignant lesion on the pancreatic body on the basis of CT scan but sooner or later intraoperative histopathologaical final results revealed noncancerous lesions in all subjects. It is unlikely that any of the 20 patients had been diagnosed with type 2 diabetes mellitus before operations with the exception of just one who was suffering from depressive disorders and was using antidepressive medications once in a while.

### 2.1. Surgical Technique

Ten patients were managed by means of midline incisions, ten through left subcostal incisions; four of them were operated for gallstones disease via open approach, so we went for midline incision in course to prevent wound dehiscence and assist in apparent anatomical view for resection.

**Figure 1:**
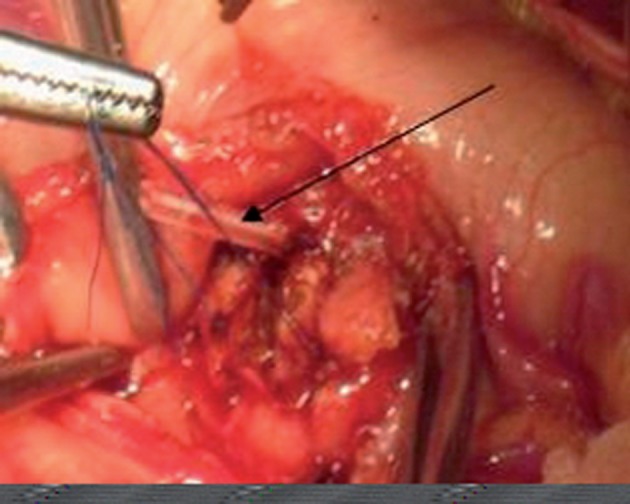
Picture illustrating the pancreatic stent passing through the post. Abdominal wall. Stent had been placed to the main pancreatic duct to prevent leakage from anastomotic site and keep patency of pancreatic juice to the stomach

The dissection begins with division of the gastrocolic ligament with entry into the lesser sac. Small vessels were ligated by 4-0 silk suture and whereever it was required, coagulation was utilized. The pancreas was then scrutinized with intraoperative ultrasonography (VIVID 4) for recognition of the neoplasm and specifying its relationship to the portal vein and superior mesenteric vein. The pancreatic dissection was performed inferiorly along the superior mesenteric vein. A tunnel was produced at the pancreatic neck; splitting the parenchyma from the posterior superior mesenteric vein/portal vein confluence. The cephalad aspect of the pancreas was then dissected from the posterior meandering splenic artery. Using four stay sutures in the cephalic and caudal parenchyma for hemostasis, the pancreas was then divided between stay sutures to the right of the lesion. The stump of the neck of the pancreas was oversewn with polypropylene suture in continuous running fashion. The proximal pancreatic duct was not oversewn independent of the main body of the pancreas ( Figure 1 ).

The body and tumor were then elevated from the posteriorly coursing splenic artery and vein. Many small tributaries were divided during this dissection with clips and fine polypropylene ligature. After accomplishing a satisfactory distal border, two additional haemostatic stay stitches were inserted in the distal remnant parenchyma, and the pancreas was divided into the left side of the tumor. Dissected sections were attained in all cases for pathological analysis to rule out the nature of the tumor and to make a distinction of its attributes. The posterior gastric wall was then added adjoining to the distal remnant and a two layer anastomosis was designed. The inner layer was designed with absorbable monofilament suture in a continuous running fashion along the circumference of the pancreas. The outer layer was constructed with fine silk sutures in interrupted fashion, causing invigilation of the cut surface of the pancreatic remnant. A pancreatic duct stent was used. Two drainage tubes were put in place in all cases, one to the anastomotic site and another just adjoining to anastomotic site to rule out any postoperative bleeding.

### 2.2. Intra-operative Findings

Twenty CP were carried out within standard anesthesia. Prior to induction of anesthesia, there was an attempt to accomplish a smooth induction. There were no intra-operative complications, with the exception of one experienced slight hypotension and there was not any blood transfusion in all cases. Operative mortality was zero. The mean anesthesia time ± SD was 313 ± 96 minutes, and the mean operative time ± SD was 222 ± 58 minutes. The mean operative blood loss ± SD was 690 ± 510 mL. Two patients underwent CP with excision of the small cyst at the right lateral and left lateral lobe of the liver correspondingly. Cystic resection and hemostasis had been done by fine prolene suture. A left flank incision for transitional cell carcinoma. In all cases, the spleen had been preserved. Mean volume of resection SD was 35.7-20.4 cm3. The mean greatest dimension of tumor ± SD was 2.6 ± 1.3 cm. The tumor size was evaluated by Vivid 4 ultrasonogarpy device. All resected tumors were sent for pathological analysis and confirmed to be cystic neoplasm of various types after histopathological evaluation ( Table 2 ).

**Table 2:** Operative characteristics of all patients

Patients	Incision Choice	Anesthesia Time(mins) median	Operation Time(mins) median	Blood Loss(ml) median	Size of Re section(cm) median	Tumor Size(cm) median	Tumor Pathology
**1**	LSC	230	170	755	14.5	1.2	Serous oligocystic adenoma
**2**	Midline	240	185	690	12.6	1.0	Mucinous cystadenoma
**3**	Midline	255	210	735	11.7	1.1	Serous microcystic adenoma
**4**	Midline	245	205	640	43.8	3.2	Mucinous cystadenoma
**5**	Midline	260	220	705	38.6	2.9	Serous microcystic adenoma
**6**	LSC	255	190	800	44.8	3.6	Neuroendocrine/glucagonoma
**7**	Midline	250	215	650	30.8	2.9	Mucinous cystadenoma
**8**	Midline	310	270	900	49.5	3.4	Mucinous cystadenoma
**9**	Midline	280	235	995	50.6	4.0	Mucinous cystadenoma
**10**	Midline	265	220	755	47.3	3.8	Mucinous cystadenoma
**11**	LSC	215	175	680	15.6	1.7	Serous oligocystic adenoma
**12**	Midline	310	275	1050	85.3	5.3	Serous microcystic adenoma
**13**	Midline	295	250	895	63.9	5.0	Serous microcystic adenoma
**14**	Midline	310	270	765	45.7	3.9	Mucinous cystadenoma
**15**	LSC	355	310	1100	69.4	4.3	Mucinous cystadenoma
**16**	Midline	380	340	1060	70.5	5.6	Serous microcystic adenoma
**17**	Midline	354	305	900	39.2	3.2	Serous microcystic adenoma
**18**	Midline	340	295	980	37.9	3.0	Mucinous cystadenoma
**19**	Midline	265	210	865	43.9	3.6	Mucinous cystadenoma
****20****	Midline	270	224	700	22.8	1.8	Mucinous cystadenoma

### 2.3. Post-operative Results

Twenty CP with PG were performed, with zero mortality (95% CI 0 to 25%) and 25% morbidity (95% CI 12.9to 43.7%). Postoperative complications incorporated 4 substantial pyrexia with urinary tract infections and two were readmitted for pancreatitis. There was no pancreatic leakage at anastomotic site. We supervised all the patients with contrast USG who complained of severe abdominal pain during postoperative period to ascertain there is not any hemorrhage from anastomotic site. Fortunately, no cases had such a problem. Intraoperative closed suction drainage catheters were taken off when it approached 40 ml or less per day, following resumption of oral soft food consumption. The mean length of perioperative drainage ± SD was 4.8 ± 1.9 days (ranging from 3 to 8 days). Two patients had drainage fluid sampled for amylase estimation because of high initial postoperative volumes but we found there is less secretion of amylase (30IU/L) comparable to amylase levels in the serum. The considerable release was due to the left disinfectant agent which came out through the drainage tube. It could possibly be due to inappropriate suction prior to concluding the abdominal wall or in obese patients there is considerably excess fat and suction of all disinfectant is rather challenging owing to substantial unwanted fat content material.

Closed-suction water flow and drainage tubes were taken out in all patients ahead of being allowed to go devoid of any discharge relevant additional complications.

Perioperative somatostatin was administered subcutaneously to all patients for a mean ± SD of 2.7 ± 1.4 days (ranging from1 to 6 days). Median lengths of hospital stay and postoperative stay were 7.5 days (ranging from 6 to 17 days; mean ± SD, 8.0 ± 3.6 days, 7.9 ± 3.4 days, respectively). It is unlikely that any of the 20 patients were aware of the clinical symptoms of exocrine insufficiency, but 2 developed late onset glucose intolerance as noticeable by elevated glycosylated hemoglobin levels. There were no cancerous growth recurrences with the median follow up of 19 months (ranging from1 to 39 months).

### 2.4. Post-operative Follow-up

**Figure 2:**
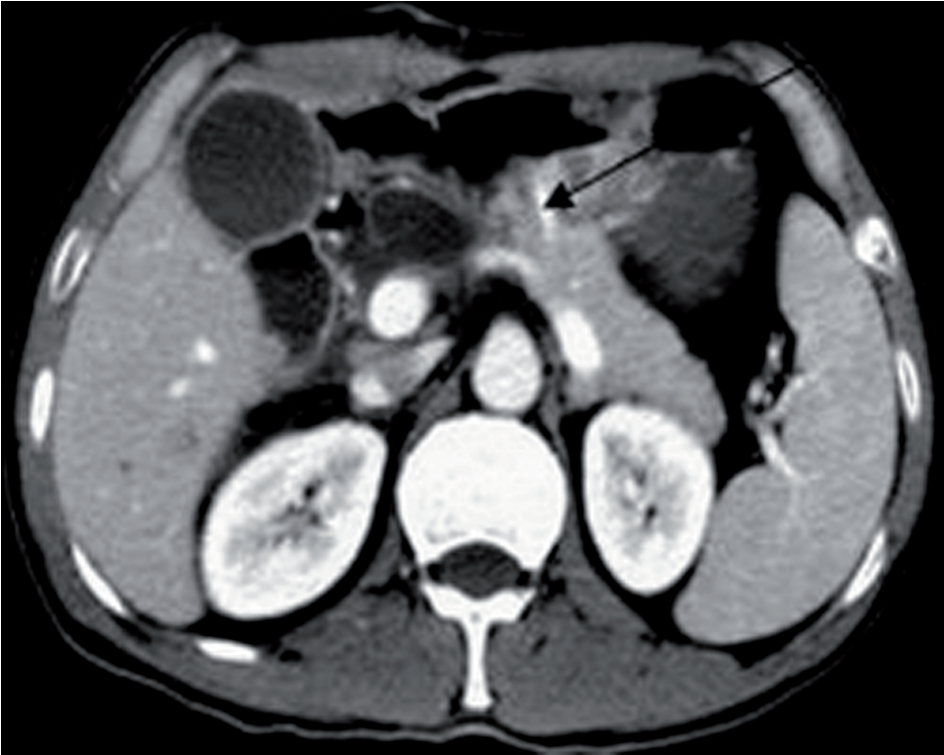
Post-operative follow-up CTscan showing the stent which had been placed to the main pancretic duct and anastomosed to the gastic wall

Of the 20 patients, 2 developed glucose intolerance during the follow up period. This diagnosis was made by identifying borderline elevated glycosylated hemoglobin levels. In either instances, the tumors were 5.6 and 4.3 cm in size, and the volumes of resection were comparatively little substantial as effectively as the affected individual acquired chronic pancreatitis. We deemed that perhaps because of the reduction of the significant section of the pancreatic remnant and chronic pancreatitis was the related variables for that. In our research, we integrated complete blood profile preoperatively to rule out if any individual has diabetes and coagulation disorders or any sort of viral or microbial infection. We noticed one most intriguing issue; two patients were discharged early in comparison to other patients. Considering that all guidelines ended up within our control although they discharged from hospital. But in the course of follow up, they showed pancreaticogenic type 2 diabetes. In our study, all patients had regular follow up ( Figure 2 ). Two patients reported abdominal distension right after taking heavy and greasy foods, having burning up feeling beneath the xiphiod process once in a while. Endoscopic examination exposed and ruled out gastric ulcer.

We interrogated for the affiliated variables of ulcer; following a comprehensive track record they explained that following the surgical treatment they drank and smoked cigarette on a regular basis. We labeled may be alcoholic beverages and cigarette smoking qualified prospects of gastric ulcer. Triple antibiotics therapy was prescribed for three weeks and they were advised to give up hot and spicy foodstuff and alcoholic beverages even more. Rest of the patients experienced ordinary daily life and probably none of them reported any type of complaint which we needed to pay particular attention.

## 3. Discussion

In the 21st century, technological improvement and excellent preoperative and postoperative critical care are foremost most highly processed consequences of post-surgical outcomes. In our analysis, pancreaticogastrostomy minimized the morbidity and mortality when there was the concern of central or distal pancreatectomy for benign tumors. On the other hand, in the current study, basic safety of PG was predominantly based on technological factors of the pancreaticogastric anastomosis. The so-called joining procedure in which a small posterior-wall gastrotomy linked with a running second stratum, which sways the gastric mucosa to the pancreatic serosa, constituted the key technological determine to protect against pancreatic leaking. Earlier postoperative hemorrhage after PG can come about frequently from the anastomotic site or from the trim borders of the pancreas. This complication constantly necessitates relaparotomy to regulate the hemorrhaging vessels (3, 4). PG revealed an additional advantageous early on outcome than PJ. PG is encouraged for specialists who come across challenges with PJ for renovation following PD. Having said that, our study was primarily conducted to determine the practicality and surgical consequences of PG. Yeo et al. conducted the very first prospective randomized study of PG vs PJ. They documented that the likelihood of PF was 12.3% in the PG group and 11.1% in the PJ group and that PG did not minimize the incidence of PF when compared to PJ. However, they have not used a telescoped or invaginated technique (5). Watanabe et al. publicized in the Japan Pancreas Surgery Group survey of 511 pancreaticogastrostomy and 2483 pancreaticojejunostomy patients. There had been no substantial dissimilarities between pancreaticogastrostomy and pancreaticojejunostomy with admiration to the occurrence rates of intrabdominal hemorrhage and abscess or mortality (6). The approach of PG has a number of prospective positive aspects in excess of PJ. Very first, the PG anastomosis can be conducted effortlessly due to the fact that the posterior wall of the stomach lies right away anterior to the mobilized pancreatic remnant and is commonly broader than the transected pancreas. Secondly, with PG, the pancreatic exocrine secretions get into the most likely acidic gastric setting, precluding the disgestive system deterioration of the pancreatoenteric anastomosis by activated proteolytic enzymes. In comparison with PJ, the triggering of pancreatic exocrine secretions can come about much more easily in the existence of intestinal enterokinase and bile. Thirdly, PG stays away from the long jejunal loop where the pancreatobiliary secretions gather for the duration of the very early postoperative period of time. Moreover, postoperative gastric decompression can offer continuous elimination of pancreatic and gastric secretions preventing deposition and consequently tension on the anastomosis. Finally, PG anastomosis lowers the quantity of anastomoses in one loop of the maintained jejunum, which most likely diminishes the probability of loop kinking. The scientific studies have preferred PG over PJ despite the fact that these scientific studies are constrained by their modest patient communities (7, 8).

Central pancreatectomy with pancreaticogastrostomy renovation is safe and sound and from a technical perspective it ought to be regarded as a secure reconstruction approach following central pancreatectomy for noncancerous ailment. Duct-to-mucosa pancreaticogastrostomy may possibly be a harmless and efficient approach for protecting against pancreatic fistula progression immediately after distal pancreatectomy when executed by knowledgeable specialists who are competent in this procedure.
